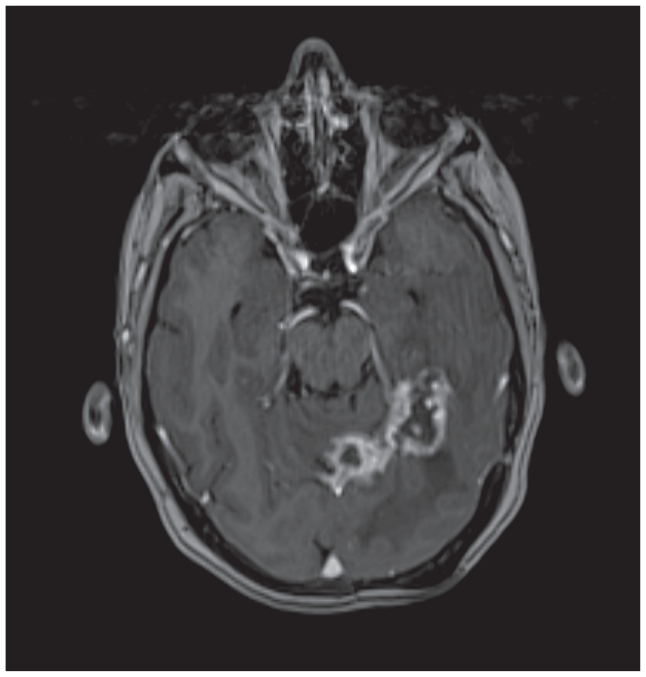# Long Survival in a Patient with Brain Metastases from Breast Cancer

**DOI:** 10.4137/cmo.s317

**Published:** 2008-02-09

**Authors:** Sanna G, Petralia G, Cossu Rocca M, Marenghi C, Nolè F

**Affiliations:** 1 Department of Medicine, Unit for Medical Care, European Institute of Oncology; 2 Division of Radiology, European Institute of Oncology

**Keywords:** brain metastases, treatment, advanced breast cancer

## Abstract

The incidence of brain metastases (BMs) is apparently rising in patients with advanced breast cancer, possibly due to better therapeutic approaches for control of metastatic growth in other organs. Occurrence of BMs severely affects quality of life and is associated with dire prognosis. In this short report we describe the clinical case of a 47 year old woman, with BMs from breast cancer diagnosed in May 2001. The patient was treated with whole brain irradiation and radiosurgery, with initial control of BMs. Due to previous radiotherapy fields and doses, further local treatments are not feasible anymore. Since September 2006, the patient has been receiving systemic therapy with Lapatinib at the dose of 1500 mg/die continuously, with a good control of cerebral, liver and nodal metastasis after one year of treatment (September 2007). Her quality of life is acceptable, her Karnofsky Performance Status (KPS) is more than 70%, and she takes care of her family, and has not experienced neuro-cognitive dysfunction.

## Introduction

The incidence of brain metastases (BMs) seems to have increased over the past decade, and may be the paradoxical result of the effectiveness of drugs that do not cross the blood—brain barrier (BBB). As a result of the increased survival in patients receiving chemotherapy, BMs may become symptomatic (1). Occurrence of brain relapse severely affects quality of life and is associated with extremely dire prognosis. Median survival after the diagnosis of symptomatic central nervous system (CNS) involvement is roughly 4 months, and 2-year survival is less than 2% (2, 3). When looking at radiosurgery series, a longer survival has been observed in selected groups of patients, with median survival ranging from 13 to 19 months (4, 5). In a recent report (6), the median survival after diagnosis of symptomatic cerebral metastases in 72 patients with metastatic breast cancer treated in our Insitution was nine months.

Breast carcinoma is the second most common cause of BMs, which occur in approximately 10%–15% of patients, although autopsy data suggest a higher prevalence up to 30% of patients (2, 7). Furthermore, breast cancer (BC) is the most common solid tumour to exhibit leptomeningeal colonization (8, 9).

A strong correlation with paclitaxel-based chemotherapy and cerebral relapse has previously been observed (1). Patients with endocrine unresponsive tumours seem at increased risk of developing cerebral metastases (1, 2). Recently, it has also been reported a higher risk of developing brain recurrence in young patients with Her-2/neu over-expression (10, 11), and in patients treated with trastuzumab-based therapy, with observed incidence rate variable from 25% to 34% (12, 13). The mechanism postulated is that Trastuzumab therapy may selectively destroy non BMs, prolong overall survival, therefore allowing a later development of cerebral relapse.

Patients with Her-2/neu over-expressing tumours had apparently a longer survival after the occurrence of BMs, compared with patients whose cancer did not express Her-2/*neu*. This survival advantage may be possibly explained by a better control of extra-cranial disease in this subgroup of patients, due to trastuzumab-based treatments (13). Data are not consistent in all reports (14), but in several studies the majority of patients with BMs and visceral disease died of progressive extra-cranial disease (15, 16).

Recursive partitioning analysis (RPA) of data from three Radiation Therapy Oncology Group (RTOG) trials (1200 patients) has allowed three prognostic groups of patients with BMs to be identified. RPA class was initially developed to categorize patients treated with fractionated external beam brain RT and tested in the radiosurgical treatment of BMs (17). Data have been validated in subsequent series (18), and RPA classes are extremely useful in estimating median survival of patients with brain recurrence according to different variables. More recently, Lorenzoni et al. proposed a simplified stratification system that uses the evaluation of three main prognostic factors for radiosurgery in BMs; this system was called of basic score for BMs (BS-BM), and may be calculated by adding the scores (0 or 1) of three main prognostic factors: Karnofsky Performance Status (KPS), control of the primary tumor, and existence of extracranial metastases, ranging from 0 (worst condition) to 3 (best condition) (19).

## Case Report

In this short paper, we describe a 47 years old woman with advanced breast cancer and brain relapse diagnosed in May 2001.

The patient underwent right mastectomy plus ipsi-lateral node dissection for breast tumour on the 19th of May 1995. Histopathological diagnosis was of high grade ductal infiltrating carcinoma, staging pT3 pN1 (4 out of 13 nodes involved) M0. Biological characteristics were as follows: negative oestrogen and progesteron receptors, ki 67 of 20%, and HER2/*neu* over-expression determined by immuno-histochemistry. She received adjuvant chemotherapy with doxorubicin (75 mg/m^2^) × 4 cycles, followed by standard CMF day 1˚ and 8˚ q 28 given endovenously for 4 cycles and complementary radiotherapy.

In September 1996, for a subcutaneous and axillary recurrence, she underwent pre-operative high-dose chemotherapy with Ifosfamide plus Cisplatin and Etoposide, and in March 1997 she performed residual lymph-node dissection with diagnosis of nodal metastasis in 3 out of 4 nodes. She then received further ‘adjuvant’ systemic treatment with Cisplatin (at the dose of 90 mg/m^2^ day 1/q21) and 5-fluorouracil given as continuous infusion (dose of 200 mg/m^2^) and radiotherapy in the axilla. In June 1998, she developed liver and nodal metastases. She was treated with Paclitaxel at the dose of 90 mg/m^2^ as a 96 h infusion in association with Vinorelbine at the dose of 15 mg/m^2^ day one and five every 21 days, resulting in complete clinical response. In April 1999, for liver progression (apparently isolated metastasis) she underwent intra-arterial hepatic chemotherapy with Docetaxel, with a complete clinical response, but few months later, in December 1999 she presented with liver and nodal recurrence.

She first underwent VI segment liver resection, and considering response to previous treatments, she started again systemic therapy with Paclitaxel (96 h infusion) and Vinorelbine for further 4 cycles, with initial response. From the second cycle, she added weekly Trastuzumab, with achievement of a clinical complete response. She continued Trastuzumab until May 2001, when she developed symptomatic BMs. Brain MRI showed the presence of 3 lesions, two located in the left cerebellum, the major with diameter 2.3 cm × 1.7 cm, the third in the right parietal lobe, with diameter of 2 cm.

Imaging showed persistence of response in extra-cranial sites of disease. In June 2001 she received whole brain irradiation with linear accelerator (20 Gy in 5 fractions), with a clinical partial response, and subsequently she underwent gamma-knife (15 Gy) of the two residual lesions (one in the left cerebellum, and one in the right parietal lobe) with achievement of a complete response.

At the end of radiotherapy in August 2001, she started again maintainance treatment with Trastuzumab until 25th of February 2002 (persistence of visceral and cerebral complete response). She then discontinued treatment and she was free of progression until August 2004, when brain CT scan showed a cerebellar lesion, apparently isolated. Considering the peculiar evolution, and the patient’s preference, she was treated with stereotactic radiosugery in September 2004 (DS 20 Gy e DM 40 Gy) with a partial response and subsequently with surgical resection of the remaining lesion to ride-termine the biological characteristics of the tumour. The histopathological diagnosis was consistent with that of the primary tumour: BMs attributable to breast origin, with oestrogen and progesterone receptor negative and Her-2/neu over-expression in more than 95% of tumour cells, determined by immuno-histochemistry.

In January 2006 brain imaging showed cerebral progression (single parietal metastases), and the patient was treated on the 8th of February 2006 with gamma-knife (15 Gy). As expected, in the following months, cerebral disease progressed again (with persistence of complete response in the visceral sites of disease). Brain imaging showed the appearance of a cerebellar mass in the site of previous radiosurgery, and surgical resection.

**Figure 1:**
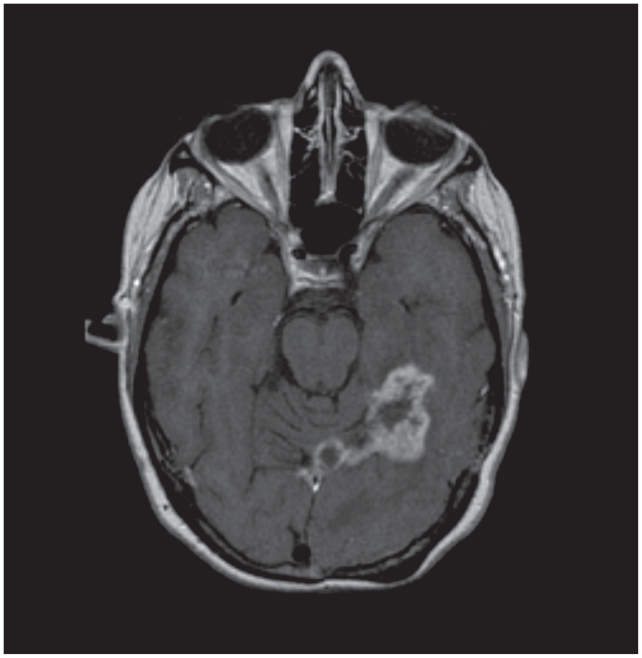


A savage systemic treatment with Carboplatin (AUC 4, on day 1) plus Gemcitabine at the dose of 1000 mg/m^2^ day 1 and 8, every 21 days, and trastuzumab (at a weekly schedule for three cycles, and with tri-weekly administration for further 3 cycles) was then started but the therapy was ineffective and cerebral disease worsened again (Fig. 1 → Axial post-contrast T1-weighted MR image obtained before treatment with Lapatinib -September 2006- shows BMs as enhancing mass, involving cranial lobe of cerebellum, temporal and occipital lobe, with longest diameter of 48.6 mm). From the 15th of September 2006 she started treatment with Lapatinib, an oral tyrosine kinase inhibitor with potent anti-Erb1 and Erb2 activity, at the dose of 750 mg bid daily (total 1500 mg) in the context of a Multicentric Phase II trial including patients with HER2/neu over-expressing advanced breast cancer pre-treated with Trastuzumab, and with progressive BMs. The treatment was well tolerated, with no G3 related toxicity. The patient complained of mild fatigue, G2, visual disturbances, and cutaneous rash (G2). All the symptoms improved modifying the schedule of administration (1500 mg once per day).

On the 5th of June 2007 a PET total body including the study of the brain was negative with no evidence of metabolic activity in the sites of disease.

She recently performed a neck, thorax and abdomen CT scan on the 24th of September 2007 which confirmed the persistence of complete visceral response (liver or nodal). A brain MRI (18th of September 2007) showed dimensional stability of cerebral lesions, with no occurrence of new sites of disease (Fig. 2 → Axial post-contrast T1-weighted MR image obtained after treatment with Lapatinib -September 2007- shows longest diameter of 47.8 mm for the BMs, which is stable disease, according to response evaluation criteria in solid tumors (RECIST).

The patient has an acceptable quality of life, her KPS is more than 70%, she takes care of her family, and has not experienced neuro-cognitive dysfunction. She is not receiving other supportive treatments (i.e. cortisonic therapy) with the exception of ranitidine 300 mg/die.

## Discussion

In our experience, we have rarely seen such a long survival in a patient with brain relapse from breast cancer and in presence of visceral involvement. This observation is of peculiar value if we consider that the patient belongs to RPA class II, with a described median survival ranging from 3.8 months to 4.2 months and a 1-year survival ranging from of 12% and 16% (18, 17).

In a recent report (6), the median survival after diagnosis of cerebral metastases in 72 patients with metastatic breast cancer treated in our Insitution was nine months. In this paper we observed a significantly higher rate of negative oestrogen receptor, negative progesterone receptors, HER2/*neu* over-expression and immunostaining for Ki67 ≥ 20% in the univariate analysis, comparing the study group with the control cohort. At multivariate analysis, HER2/*neu* over-expression and Ki67 ≥ 20% were independent predictive factors of brain relapse and endocrine unresponsive tumours (both ER and PgR < 10%) showed an increased risk of central nervous system recurrence of borderline significance.

In tumours over-expressing Her2/neu, BMs seem to mantain the biological phenotype. The clinically important obstacle of delivering trastuzumab to an intracerebral metastasis was addressed by Grossi et al. (20). These investigators modeled HER2-overexpressing BMs in athymic rats. They implanted a human breast cancer cell line that overexpressed HER2 into the brain by intracerebral injection. The rats were treated with Trastuzumab or a control, isotype-matched antibody using a microinfusion technique that utilizes bulk flow current (convection) to deliver large proteins into the brain. Trastuzumab or the control antibody was administered regionally via an intracerebral cannula directly into the tumor for 7 days. Animals treated with intracerebral microinfusion of Trastuzumab had significantly improved survival compared with controls. However, systemic administration of Trastuzumab via i.p. microinfusion failed to deliver the drug to the brain and did not significantly affect survival. These results demonstrate that Her-2/neu over-expressing breast cancer, which is growing in the brain, can be targeted with HER2-directed therapy if the drug penetrates the CNS (20).

Lapatinib is an oral receptor tyrosine kinase inhibitor, inhibiting both the ErbB-1 and ErbB-2 receptors. Lapatinib has been shown to have activity in ErbB-2-overexpressing breast cancer in several phase II and III clinical trials. Specifically, Lapatinib is effective in patients with metastatic breast cancer, with inflammatory breast cancer, and possibly, with BMs (21).

Clinical trials have been carried out with Lapatinib for the treatment of BMs. Results from a phase II trial with Lapatinib for Her-2/neu over-expressing breast cancer patients with new or progressing BMs were presented at the 2006 American Society of Clinical Oncology (ASCO) Annual Meeting. (22). In that study, patients received oral Lapatinib at a dose of 750 mg twice daily. The primary end point was objective response in the central nervous system. Thirty-nine patients who had developed CNS disease during treatment with Trastuzumab were enrolled. By the Response Evaluation Criteria in Solid Tumors (RECIST), two patients (5%) had a partial response (PR) as the best CNS response and 4 of 16 patients (25%) with measurable disease had a PR as the best non-CNS response. Eight patients had stable disease (SD) in the CNS at 16 weeks. Lapatinib was well tolerated in this patient population and there was some evidence of CNS clinical activity.

## Conclusions

The design of therapeutic and preventive approaches to brain relapse would further benefit from an increased understanding of the blood-brain and blood-tumour barriers as well as other host-tumour interactions in the CNS. In high risk patients, the use of adjuvant therapies potentially active against cerebral metastases (Lapatinib) should be prospectively investigated.

## Figures and Tables

**Figure 2:**